# TASPERT: Target-Specific Reverse Transcript Pools to Improve HTS Plant Virus Diagnostics

**DOI:** 10.3390/v13071223

**Published:** 2021-06-24

**Authors:** Andres S. Espindola, Daniela Sempertegui-Bayas, Danny F. Bravo-Padilla, Viviana Freire-Zapata, Francisco Ochoa-Corona, Kitty F. Cardwell

**Affiliations:** 1 Institute of Biosecurity and Microbial Forensics (IBMF), Oklahoma State University, Stillwater, OK 74078, USA; daniela.sempertegui.2013@gmail.com (D.S.-B.); dannyfco.99@gmail.com (D.F.B.-P.); freirez@okstate.edu (V.F.-Z.); ochoaco@okstate.edu (F.O.-C.); kitty.cardwell@okstate.edu (K.F.C.); 2 Department of Entomology and Plant Pathology, Oklahoma State University, Stillwater, OK 74078, USA

**Keywords:** HTS diagnostics, TASPERT, template switching oligo, MiFi, microbe finder, HTS, diagnostics, tobacco ringspot virus, TRSV, sensitivity, sequencing

## Abstract

High-throughput sequencing (HTS) is becoming the new norm of diagnostics in plant quarantine settings. HTS can be used to detect, in theory, all pathogens present in any given sample. The technique’s success depends on various factors, including methods for sample management/preparation and suitable bioinformatic analysis. The Limit of Detection (LoD) of HTS for plant diagnostic tests can be higher than that of PCR, increasing the risk of false negatives in the case of low titer of the target pathogen. Several solutions have been suggested, particularly for RNA viruses, including rRNA depletion of the host, dsRNA, and siRNA extractions, which increase the relative pathogen titer in a metagenomic sample. However, these solutions are costly and time-consuming. Here we present a faster and cost-effective alternative method with lower HTS-LoD similar to or lower than PCR. The technique is called TArget-SPecific Reverse Transcript (TASPERT) pool. It relies on pathogen-specific reverse primers, targeting all RNA viruses of interest, pooled and used in double-stranded cDNA synthesis. These reverse primers enrich the sample for only pathogens of interest. Evidence on how TASPERT is significantly superior to oligodT, random 6-mer, and 20-mer in generating metagenomic libraries containing the pathogen of interest is presented in this proof of concept.

## 1. Introduction

Plant quarantine agencies worldwide have shown interest in adopting HTS as a diagnostic tool [1,2,3]. Barriers to fully use HTS as a plant diagnostic tool are the price and sensitivity of the technique. However, the advent of new HTS platforms such as MinION from Oxford Nanopore Technologies (ONT) has driven down the cost of sequencing, contributing to portability and the capacity to stop sequencing when the diagnostic target is found [4]. These are valued characteristics that make MinION an attractive choice for diagnostics. MinION has been used to detect multiple human and animal viruses worldwide [5,6], including a proof of concept for plant virus diagnostics [7]. Competing technologies such as Illumina offer short reads with decreased error rates, yet the equipment is costly and lacks the portability feature. Regardless of the pros and cons of these two available HTS platforms, effective pathogen detection depends on high-quality samples with sufficient pathogen DNA/cDNA. Samples with pathogen DNA/cDNA in low concentration require deeper sequencing, resulting in increased costs. For example, some RNA plant viruses have shown to have varying concentrations in the host depending on season, days after inoculation, and other abiotic factors [8]. When plant RNA viruses are in low concentration, their detection becomes challenging with any molecular technique, including HTS.

Many methods that aim to increase or enrich the target RNA/cDNA have been described, yet these are often time-consuming and expensive [9]. The SMART method was first described in 2001 by Zhu et al. [10]. It relies on an intrinsic characteristic of the Moloney murine leukemia virus (MMLV) reverse transcriptase. MMLV is capable of synthesizing an anchor of non-templated cytosine nucleotides at the 3′-terminus of the first-strand cDNA. The anchor is subsequently used to synthesize the second strand of the cDNA generating a full-length cDNA transcript. The technique has been used for single-cell RNA-seq in multiple studies [11,12,13,14]; however, it has never been applied for HTS diagnostics. We had hypothesized that incorporating the SMART method into an HTS-based diagnostic pipeline allows to specifically enrich predetermined targeted pathogens, leading to a larger number of mapped reads and deeper pathogen sequencing. We are naming this procedure TArget-SPecific Reverse Transcript (TASPERT) pools. TASPERT uses pathogen-specific reverse primers/probes coupled with the SMART system to enrich the pathogen of interest. Once the primers are validated for each targeted RNA virus, the reverse primers are pooled. The pool of primers is used to generate double-stranded cDNA from known or pre-determined positive and negative control samples. This study demonstrates that combining SMART technology with a specifically developed pool of RT primers targeting the 3′-terminus of the pre-determined pathogens increases the pathogen abundance up to twenty times more than when using the traditional poly(T)/oligodT primer or random primer approaches. TASPERT allows obtaining HTS data enriched for any RNA virus of interest. The data can be screened with Microbe Finder (MiFi) or any other bioinformatic software to determine the presence of pathogens in samples of interest [15,16,17] with increased statistical significance.

## 2. Materials and Methods

### 2.1. Experimental Design

Two viruses were selected for this experiment because of their different genome organization and composition. Tobacco ringspot virus (TRSV) is a bi-partite + ssRNA virus with poly(A) tail at the 3′-terminus. In contrast, grapevine leafroll associated virus 3 (GLRaV-3) is a monopartite +ssRNA virus without poly(A) tail at the 3′-terminus. The TASPERT pools protocol was compared with the other three protocols widely used for ds-cDNA synthesis that include oligodT and two lengths of random primers (6-mer and 20-mer). Double-stranded cDNA was synthesized using the four techniques for TRSV and GLRaV-3 replicated 12 times. A total of 96 Oxford Nanopore MinION libraries were prepared for the purpose of this experiment, 48 for each virus.

### 2.2. Samples and RNA Extraction

Grapevine tissue infected with TRSV and GLRaV-3 was sourced by Cornell University. Total RNA extractions of samples were conducted using the Qiagen RNeasy^®^ Plant Minikit (Hilden, Germany) (Cat# 74094) with modifications that included the addition of 0.04 g of polyvinylpyrrolidone (PVP) to 1 mL buffer RLT. The pH of the modified RLT buffer was adjusted to 5.0–5.5 using 0.2 M sodium acetate, and the final solution was kept on ice until use. Small pestles that fit into 2 mL tubes were used for cell disruption of the leaves. Liquid nitrogen was used throughout the cell disruption process. The rest of the protocol was performed following the manufacturer’s guidelines. The purified RNA was kept at −80 °C until use.

### 2.3. Reverse Primer (RT Primer) Design at the 3′-Terminus for TASPERT

Primers were developed for TRSV and GLRaV-3 from 45 full genomes available in public databases (NCBI and EMBL). Multiple sequence alignment using Muscle [18] was performed with 44 GLRaV-3 genomes, and a consensus sequence was retrieved. The 3′-terminus of the consensus sequence was used as a template for primer design (RT primer) using Primer3 [19]. The selected primer thermodynamic conditions included the following: melting temperature (Tm) as default; the minimum allowed maximum Tm difference (commonly 0.5–5 °C), minimum primer GC% 20, optimum 50, and maximum 55; the minimum allowed maximum self-complementarity was set from 0 to 3 and progressively allowed to raise until 8 only if necessary; the minimum allowed maximum 3′ self-complementarity was fixed at 0 as optimum and 2 or 3 were allowed only if strictly necessary; the maximum poly-X or maximum allowable length of a mononucleotide repeats was 3 and allowed to raise only if strictly necessary. In cases where SNPs and/or high variability were found in the consensus sequence used for primer design, degenerate nucleotides were incorporated in such SNPs or variable positions. Moreover, an additional 5′ sequence extension or “flap” non-complementary to the primer target sequence was coupled to each RT primer [20]. This primer construct that allows the specific enrichment of viral ds-cDNA is named, for the purpose of this research, flap-amplification-primer (FAP) (Table 1). To ensure that the primers do not interfere with other templates or dimerize with primers in the reaction, primer-BLAST [21] and the Oligo Analyzer (IDT) were used to evaluate the RT-primers for cross-reactivity and primer-dimer. To execute this proof of concept, the template switching oligo (TSO), FAP, and amplification primers were sourced from the NEBNext Single Cell/Low Input cDNA Synthesis and Amplification Module (New England Biolabs, Ipswich, MA, USA). The RT primers containing the 5′ FAP were combined in a pool where all primers had a final concentration of 5 μM.

### 2.4. cDNA Synthesis and Amplification with TASPERT Pools

Fresh or stored RNA (−80 °C) was used. Briefly, the primer annealing and first-strand synthesis reaction mix included 4 μL of RNA (~60 ng); 1 μL of RT primer mix pool, 10 μM; and 1 μL of dNTP, 10 μM. The mix was incubated for 3 min at 70 °C and held on ice until use. The thermal cycler lid was set at 95 °C. The reverse transcription and template switching reaction was prepared by mixing 2.5 μL of Template Switching RT buffer (NEB Cat # M0466L), 1×; 0.5 μL of template switching oligo, 75 μM; and 1 μL of template switching RT enzyme mix (NEB Cat # M0466L). The 4 μL of the RT reaction mix was combined with 6 μL of annealed mix. A combined volume of 10 μL was incubated for 90 min at 42 °C, then 5 min at 85 °C and held on ice until use. Finally, the amplification process was performed by mixing 10 μL of the template switching cDNA from the previous step; 25 μL of NEBNext High-Fidelity 2X PCR Master Mix (NEB Cat # M0541); 1 μL of cDNA PCR primer, 10 μM; and 14 μL of water for a total reaction volume of 50 μL. The mix was incubated in a thermal cycler with initial denaturation at 98 °C for 45 s and 21 cycles of denaturation at 98 °C for 10 s, annealing at 62 °C for 15 s, and extension at 72 °C for 8.5 min. A final extension of 8.5 min was added after cycling. The amplification products were stored at −20 °C until use.

#### 2.4.1. Assessing the Presence of TRSV and GLRaV3 in the ds-cDNA

Quantification of the obtained ds-cDNA was performed using the Quantus fluorometer model E6150 with the QuantiFluor ONE dsDNA System (Cat # E4870). The presence of TRSV and GLRaV-3 in the obtained ds-cDNA was assessed using real-time PCR. Specific primers targeting the TRSV (TRSV1-F and TRSV1-R) and GLRaV-3 (GLRaV-3A-F and GLRaV-3A-R) previously reported were used [22,23]. PowerUp SYBR Green Master Mix (ThermoFisher Cat # A25742) was used as a fluorophore. The thermal cycling conditions for both primer pairs were 50 °C for 2 min, 94 °C for 5 min, and 35 cycles of 94 °C for 30 s, 56.8 °C for 45 s, and 72 °C for 60 s, followed by a final extension of 72 °C for 5 min. Post-qPCR HRM parameters used were pre-melting conditioning at 60 °C for 1 min and a melting temperature cycle ranging from 60 °C to 99 °C, with increments of 0.5 °C every 2 s. All the RT-qPCR-HRM reactions were performed in a Rotor-gene 6000 thermocycler (Qiagen, Valencia, CA). The melting curve normalization was computer-assisted using Rotor-Gene Q-Series Pure Detection Software v. 2.3.1.

#### 2.4.2. Sequencing and HTS Diagnostics

The ds-cDNAs were used to generate long-read Oxford Nanopore sequencing libraries. The Direct cDNA Native Barcoding (SQK-DCS109 with EXPNBD104 and EXP-NBD114) version DCB_9091_v109_revJ_14Aug2019 was used to barcode up to 24 samples in a single library preparation. The barcoded ds-cDNA samples were quantified before pooling using the Quantus fluorometer model E6150 with the QuantiFluor^®^ ONE dsDNA system (NEB Cat # E4870). The barcoded libraries were pooled equimolarly and sequenced in MinION using flowcell version R9.4.1. The sequencing was run using the software MinKNOW version 4.1.22 for 72 h. The base-calling was performed using the Guppy basecaller version 4.0.14+8d3226e, and demultiplexing was performed using guppy barcoder version 4.0.14+8d3226e. The base-calling and demultiplexing was done by the Oklahoma State University High Performance Computer (Pete) using a node containing an NVIDIA Quadro RTX6000 GPU. Mapping the reads to reference genomes of GLRaV-3 (AF037268.2) and TRSV (U50869.1 and AY363727.1) was performed using minimap2 version 2.10-r761. To determine if any bias exists in the depth of coverage towards the 3′-terminus when using the TASPERT protocol, Samtools version 1.10 [24] was used to calculate the depth of coverage at different coordinates of the TRSV and GLRaV-3 genomes. The detection of TRSV and GLRaV-3 was done using the Microbe Finder (MiFi^®^) online system [15] using e-probes that have been previously developed and validated for TRSV and GLRaV-3. The RT primers (RT-primer control) were aligned to each of the generated metagenomes using the BLASTn algorithm to determine potential cross-contamination. The use of the RT primers as control added confidence to the demultiplexing process to determine sources of contamination during library preparation.

## 3. Results

### 3.1. Reverse Primer (RT Primer) Design at the 3′-Terminus for TASPERT

**Figure 1:**
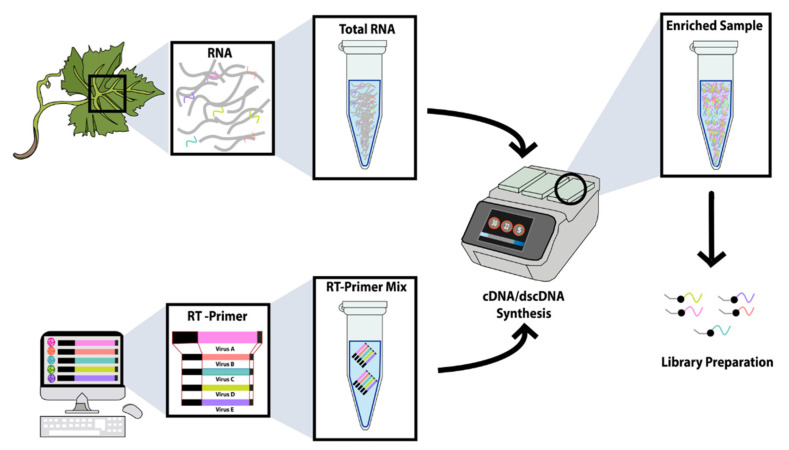
TASPERT method layout showing the sample RNA extraction from infected tissue (**top left**), the RT-primer design (**bottom left**), and the virus-specific ds-cDNA synthesis (**right**). Short colored strands in the RNA extraction section represent viral RNA from different plant viruses, while longer gray strands represent host RNA. FAP are colored black at the 5′ of the RT-primers, and the virus-specific segments are represented with different colors. A degenerate nucleotide representing (A/C/G) is also represented in black at the 3′-terminus of the RT-primers. Only the virus sequences are enriched during ds-cDNA synthesis using the TASPERT RT-primers (**center-right**). The final TASPERT product is ready for library preparation and sequencing with any HTS platform.

The length of the RT-primers ranged from 44 to 45 nucleotides including the 5′ FAP (Figure 1). Their mean melting temperatures were 62.7, 65, and 65.2 °C for dTRSVTSO, dTRSVTSO2, and TSO-GLRaV-3-a, respectively. The TSO and amplification primer melting temperatures were 66.6 °C and 58.6 °C, respectively (Table 1). Homo-dimer analysis predicted secondary structures requiring delta-Gs of up to 6.84, 6, and 6.76 kcal/mole for TRSV RNA1, TRSV RNA2, and GLRaV-3 RT-primers. Their GC content was 37.9%, 41.5%, and 42.2% for dTRSVTSO, dTRSVTSO2, and TSO-GLRaV-3-a, respectively.

### 3.2. Assessing the Presence of TRSV and GLRaV3 in the ds-cDNA

The presence of TRSV and GLRaV-3 in the ds-cDNA was demonstrated by qPCR before sequencing. Ct values showed that ds-cDNA of each pathogen were present in high concentrations (Ct values lower than 23) in all tested protocols (oligodT, TASPERT, and random primers) (Table 2). The concentration of the TASPERT-generated ds-cDNA (25 ng/uL average) suggested high enrichment yield for both GLRaV-3 and TRSV metagenomes.

### 3.3. Determining Pathogen Abundance and Presence in Sequencing Library

**Figure 2:**
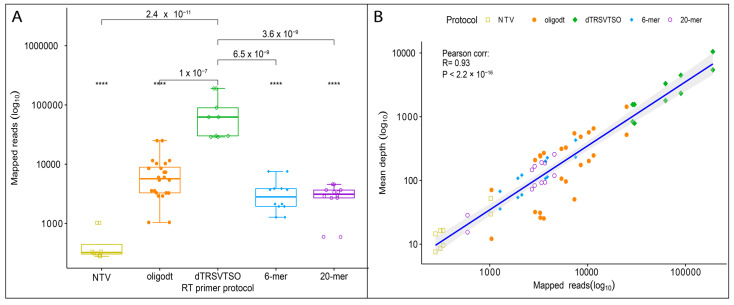
Comparison of libraries generated with TASPERT, OligodT, random 6-mer, and 20-mer RT-primers. (**A**) Number of reads mapped to the TRSV reference genome showing the highest pathogen abundance in the metagenome was achieved using the dTRSVTSO RT-primers. (**B**) Correlation between mapped reads and mean depth for TRSV showing dTRSVTSO primers allowed a larger number of mapped reads and deeper exploration during HTS of the sample. A group of non-target virus (NTV) acting as negative control was included. These NTVs did not contain TRSV but grapevine red blotch virus (GRBV). Statistical value meanings in the figure are: ****: *p* ≤ 0.0001.

All barcoded libraries generated pathogen reads during HTS regardless of the RT primer treatment (Table 2). However, the number of pathogen reads generated was significantly higher in the libraries that were prepared using the TASPERT RT-primers (Figure 2A). In some instances, the relative abundance was 21 times higher when using the TASPERT protocol. The mean sequencing depth was correlated with the number of mapped reads (Figure 2B). The average depth of coverage (mean depth) was higher with the TASPERT primers (Figure 2B); however, most of the coverage was biased towards the 3′-terminus in both pathogen genomes. Specifically, sequencing reads of the TASPERT protocol generated on average 641.21 (GenBank: AY363727.1) and 74.74 (GenBank:U50869.1) times more depth of coverage towards the 3′-terminus in the TRSV genome. The same behavior was observed in the GLRaV-3 genome, having on average 173.93 (GenBank: AF037268.2) more depth of coverage towards the 3′-terminus of the genome.

**Figure 3:**
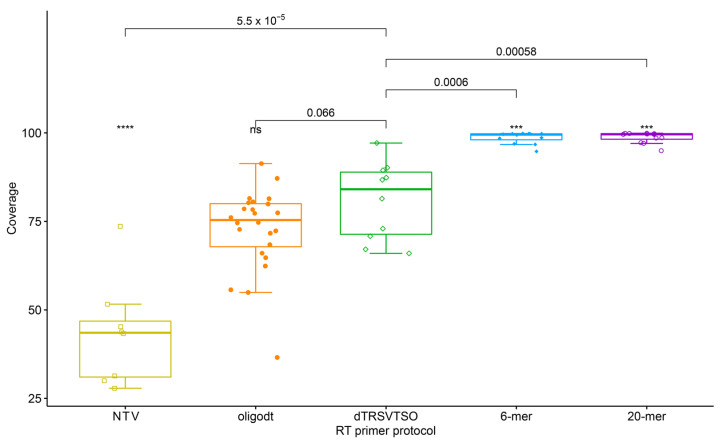
Comparison of reads mapped to the TRSV genome after HTS of libraries generated with the TASPERT, OligodT, random 6-mer, and 20-mer RT primers. NTV is the reference negative control. Statistical value meanings in the figure are: ns: not significant, ****: *p* ≤ 0.0001 and ***: *p* ≤ 0.001.

When assessing the genome coverage for each treatment, the random hexamer primers generated significantly higher coverage when compared with TASPERT and oligodT primers (Figure 3). The BLASTn search of the RT-primers in all the generated metagenomes served as a cross-contamination control. RT primers are only expected in samples that were sequenced from TASPERT-generated ds-cDNA. The RT primer search showed the primer was also found at a lower rate in samples that used the oligo-dT and random primers (Table 2).

### 3.4. Detection of TRSV and GLRaV-3

Pathogen detection in metagenomes obtained after HTS was made by bioinformatic means. Various bioinformatic methods are used to determine pathogen presence in metagenomes, which include mapping the reads to the reference genome as well as local alignment algorithms (BLAST) using large public databases. The results from the Minimap2 application suggest the pathogen presence in a sample; however, the final decision may need additional data such as the pathogen titer and season of the occurrence, which may be relevant to confirm the certainty of the finding. Alternatively, dedicated bioinformatic pipelines that use curated databases (e-probes) [15] can be used. The e-probes directly provided the result (positive or negative) for TRSV and GLRAv-3 in the sample. In this study, e-probes detected almost all GLRaV-3 TASPERT-derived samples and all TRSV samples (Table 2).

The MiFi platform determined the presence of GLRaV-3 and TRSV using pathogen-specific e-probes within the first 10 min of the sequencing run. All the samples containing GLRaV-3 and TRSV were detected with lower *p*-values than those that were generated using the non-TASPERT technique, suggesting that e-probes were hitting at an increased rate [25]. The hit numbers on the TASPERT-generated samples increased by 20×, also suggesting the use of this ds-cDNA synthesis technique could potentially decrease the Limit of Detection (LoD) of any plant pathogen to lower concentrations.

## 4. Discussion

Pathogen abundance has been a concern when using HTS for diagnostics in plants [24]. Various approaches have been explored at the molecular and bioinformatic levels and with different crops to improve the likelihood of detecting a pathogen [25,26,27,28,29,30,31,32,33,34]. The TASPERT protocol allows samples to be enriched in about 45 min before starting a library preparation intended for HTS diagnostics in plants. Primers needed for TASPERT can be developed for any pathogen, but most importantly for RNA viruses, which do not have a known generalized barcode to allow enrichment.

The effects of using specific RT-primers (TASPERT) for the ds-cDNA synthesis of TRSV and GLRaV-3 were an increased pathogen presence in the ds-cDNA, demonstrated by the Ct values of the qPCR and larger number of pathogen reads in the metagenome generated after HTS (Figure 2). The TASPERT protocol outperformed other ds-cDNA synthesis protocols in terms of pathogen read abundance in the metagenome by up to 20 times (Table 2). The increased relative abundance of the pathogen (mapped reads/total reads) in the HTS library developed from TASPERT ds-cDNA improved the likelihood of finding the pathogen using fewer sequencing resources. Specifically, the total metagenomic reads required to detect a pathogen were lower, decreasing sequencing costs and increasing the sensitivity of the assay (Table 2/Figure 2A). E-probes did not detect the pathogen when using the oligodT in GLRaV-3 samples because of the low pathogen read abundance (Table 2). The false-negative results in GLRaV-3 may also be due to a low number of e-probes. In contrast, reads mapped to the GLRaV-3 genome were abundant. E-probes detected the pathogens in the metagenome, with lower p-values in all TASPERT-generated sequencing libraries (Table 2). The significantly higher pathogen reads and high genome coverage (66–97%) suggest that any other bioinformatic tool should be able to detect TRSV or GLRaV-3 using the TASPERT-generated libraries.

The nature of the reverse transcriptase enzyme, which starts the reverse transcription at the 3′-terminus of the target RNA, creates an uneven genome coverage for any 3′-terminus RT-primer, including the widely used oligodT. The SMART technique uses oligodT and showed no significant differences in genome coverage when compared with TASPERT (Figure 3). This similarity occurs because the poly(A) tail is only a few nucleotides downstream of the specific RT-primers for TRSV. Yet, genome coverages generated by TASPERT are slightly higher than oligodT. The quality and integrity of the RNA are key to generate full transcripts. Highly fragmented RNA lowers the chances of getting long fragments, therefore reducing probative value. The fact that random oligos had higher genome coverage than TASPERT is explained by the way the TASPERT primers work. The TASPERT primers are designed in the 3′-terminus of the virus genome, and it depends on the integrity of the viral RNA to reach full-length cDNA synthesis. On the other hand, random oligos can bind in any fragmented viral RNA, producing higher coverage, but shorter and fewer pathogen reads. Additionally, random oligos will bind to the host RNA, increasing the host nucleic acid abundance in the metagenomic output. The significantly higher read abundance found in TASPERT-generated ds-cDNA was mostly biased towards the 3′-terminus; however, this is circumvented by developing RT primers every 1.5 Kb in the genome of the RNA virus. Low coverage is not an issue when HTS is used for diagnostics, though it is important for genome assembly.

The use of the TASPERT protocol can be extrapolated to other pathogens as described. Fully assembled genomes from RNA viruses are required to develop primers in the 3′-terminus. Most importantly, TASPERT allows for the detection of multiple pathogens in a single reaction because these RT-primers can be pooled. The pooling of the primers is only limited by the level of dimerization of the primers. All RT-primers were tested for dimerization and cross-reactivity. Therefore, this technique can allow pooling with a virtually unlimited number of primers. Similar primer pools of up to 1954 primer sets have been previously made for amplification of human DNA markers [35]. With TASPERT, each pathogen must be individually validated following all diagnostic performance metrics with pooled primers. Further research is needed to assess the feasibility of using TASPERT with negative-strand RNA viruses as well as DNA viruses. This proof of concept used technical replicates from the same positive controls. This was required to minimize the effect of other variables that could affect the tested hypothesis. However, when validating TASPERT for diagnostic purposes, isolates from different geographical areas should be used to generate ds-cDNA. Additionally, TASPERT can be applied in combination with other enriching techniques such as 16S for bacteria and fungi to generate a comprehensive representation of virus, bacteria, and fungi in a single step [36,37]. TASPERT ds-cDNA can be used with any type of downstream sequencing platform because similar techniques have generated full-transcript ds-cDNA [38,39,40]. The average fragment length generated after TASPERT was 1.5 Kb. Therefore, if further fragmentation is needed for Illumina sequencing or other short fragment sequencing technology, fragmentation could be performed without the risk of losing transcript titer. TASPERT is a targeted approach and therefore can only be used for diagnostics of known viruses, where fully assembled genomes are available. The limitation of using targeted approaches in HTS diagnostics is that there is a risk of not capturing the full diversity of the virus of interest. Therefore, validation using isolates from distinct geographical areas is imperative to determine the usability of the RT primers. Additionally, this targeted technique will not be useful for virus discovery unless degenerate primers are developed, taking into consideration virus evolution. Finally, this approach does not add more steps to the ds-cDNA synthesis since it is only replacing the often used random or oligodT primers with a modified protocol (TASPERT) to obtain full-length ds-cDNA for HTS.

## Figures and Tables

**Table 1:** Primer sequence sets used for ds-cDNA synthesis (RT-specific primers, template switching oligo (TSO) and amplification primer) and qPCR specific primers.

Primer Name	Type	Primer Sequence (5′ to 3′)
dTRSVTSO	RT primer RNA1	**AAGCAGTGGTATCAACGCAGAGTAC**TTAACTAGAGATTTTACTV
dTRSVTSO2	RT primer RNA2	**AAGCAGTGGTATCAACGCAGAGTAC**CTTTAGAAAACYCAAYAGAV
TSO-GLRaV-3-a	RT primer	**AAGCAGTGGTATCAACGCAGAGTAC**GACCTAACTTATTGTCGATA
TSO	TSO	GCTAATCATTGCAAGCAGTGGTATCAACGCAGAGTACATrGrGrG
TSO-PCR Primer	Amplification primer	AAGCAGTGGTATCAACGCAGAGT
GLRaV-3A-F	qPCR	TACGTTAAGGACGGGACACAGG
GLRaV-3A-R	qPCR	TGCGGCATTAATCTTCATTG
TRSV1-F	qPCR	CAGGGGCGTGAGTGGGGGCTC
TRSV1-R	qPCR	CAATACGGTAAGTGCACACCCCG

FAP primer section is underlined and bold.

**Table 2:** Double-stranded cDNA concentrations generated using the TASPERT protocol and the NEBNext Single Cell protocol. The RT primers for TASPERT were TSO-GLRaV-3-a and dTRSVTSO for GLRaV-3 and TRSV, respectively. Double-stranded cDNA was measured with Quantus, and pathogen detection was performed using qPCR, read mapping, and e-probes. The control RT primer was used to determine if cross contamination occurred.

Sample	C_t_ Value (qPCR)	Mapped Reads (Ref. Genome)	Coverage (Ref. Genome)	Sequence Depth (Mean)	Control (RT Primer)	E-Probes Detection (*p*-Value)	ds-cDNA Protocol
GLRaV-3-1-47-1t	18.52	349,668 (35.88%)	100	30,498.20	199	Positive (6.23 × 10^−5^)	TASPERT
GLRaV-3-1-47-2t	21.98	201,241 (64.79%)	87.53	14,792.60	129	Positive (2.63 × 10^−2^)	TASPERT
GLRaV-3-1-47-1n	11.21	3531 (13.63%)	98.07	207.88	4,607	Negative (>0.05)	OligodT
GLRaV-3-1-47-2n	10.96	854 (7.24%)	72.66	48.07	1,214	Negative (>0.05)	OligodT
GLRaV-3-1-47-1t	12.18	210,741 (29.13%)	100	16,192.60	4,649	Positive (8.24 × 10^−4^)	TASPERT
GLRaV-3-1-47-2t	11.1	28,345 (35.88%)	97.15	1449.09	2,788	Positive (3.36 × 10^−2^)	TASPERT
GLRaV-3-1-47-3t	12.23	30,005 (38.96%)	84.92	1243.79	3205	Negative (0.053)	TASPERT
TRSV-2-55-1a	19.94	3287 (1.03%)	71.64; 54.92	239.6; 26.03	249	Positive (0.021)	OligodT
TRSV-2-55-2a	20.05	7327 (0.90)	81.39; 62.38	548; 50.46	618	Positive (0.018)	OligodT
TRSV-2-55-3a	19.86	3570 (1.32%)	74.59; 78.54	269.54; 25.32	282	Positive (0.038)	OligodT
TRSV-2-56-1a	16.49	2905 (0.71%)	78.32; 66.02	208.49; 31.89	306	Negative (0.12)	OligodT
TRSV-2-56-2a	22.11	3268 (0.69%)	68.42; 72.31	246.77; 31.04	375	Positive (0.013)	OligodT
TRSV-2-56-3a	21.21	1042 (0.68%)	55.68; 36.55	70.82; 12.04	143	Negative (0.51)	OligodT
TRSV-2-55-1b	14.67	30,075 (16.98%)	81.41; 65.97	787.94; 1551.7	21,210	Positive (0.032)	TASPERT
TRSV-2-55-3b	15.36	89,605 (14.34%)	89.45; 72.94	2330.65; 4459.01	86,618	Positive (0.00052)	TASPERT
TRSV-2-56-1b	15.05	190,058 (14.50%)	97.13; 86.74	5418.14; 10483.7	186,645	Positive (8.17 × 10^−5^)	TASPERT
TRSV-2-56-2b	14.46	28,853 (10.09%)	87.36; 70.83	835.93; 1552.79	39,209	Positive (5.03 × 10^−3^)	TASPERT
TRSV-2-56-3b	13.58	62,481 (12.15%)	90.17; 67.12	1789.45; 3305.49	66,044	Positive (3.26 × 10^−3^)	TASPERT

n: NEBNext Single Cell protocol with TASPERT RT primers; t: TASPERT protocol with GLRaV-3 RT-primer; a: Poly(T) RT primer with TASPERT protocol; b: TASPERT protocol with TRSV RT-Primer; Ct values are the average of two replicates.

## Data Availability

The sequencing data will be made available using the SRA NCBI cloud system once this manuscript is accepted for publication.
